# Degenerative Lumbar Spine Surgeries Under Regional Anesthesia in a Developing Country: An Initial Case Series

**DOI:** 10.7759/cureus.34065

**Published:** 2023-01-22

**Authors:** Oswin Godfrey, Rabeet Tariq, Saad Akhtar Khan, Manzar Hussain, Urooba Ahmed

**Affiliations:** 1 Neurosurgery, Liaquat National Hospital and Medical College, Karachi, PAK; 2 Neurosurgery, Aga Khan University Hospital, Karachi, PAK

**Keywords:** regional anesthesia, laminotomy, laminectomy, lumbar spine, spinal anesthesia

## Abstract

Introduction

Current evidence from developed countries on lumbar spine surgeries under regional anesthesia reports it to be superior to general anesthesia (GA) in terms of decreased anesthesia time, operative time, intraoperative complications such as bleeding, postoperative complications, length of hospital stay, and overall cost. We report the first case series from Pakistan on lumbar spine surgeries under regional anesthesia.

Methods

We utilized spinal anesthesia (SA) for lumbar spine surgeries of 45 patients in a tertiary-care hospital in Karachi, Pakistan. The surgeries were performed as day-care procedures. The preoperative assessments included MRI findings, visual analogue scale (VAS), pre-operative limb powers, and straight leg raise (SLR). Other assessments included total SA time, total surgical time, time of stay in the post-anesthesia care unit (PACU), complications, and total hospital cost. SPSS v26 was used to calculate means and standard deviations.

Results

We found the total SA time to be about 45 to 60 minutes in most patients (95.6%). The total surgical time was 30 to 45 minutes for most patients. The average time of stay in the PACU was three to four hours. The VAS scores were significantly improved postoperatively with 46.7% (n=21) of patients with a score of 3, 46.7% (n=21) with a score of 2, and 6.7% (n=3) with a score of 1. 71.1% (n=32) patients had day-care surgery, 22.2% (n=10) stayed in the hospital for one day, and 6.7% (n=3) patients stayed for more than one day. Most patients (88.9%, n=40) had no complications, whereas only 11.1% (n=5) complained of PDPH. The total hospital cost was also lesser than procedures under GA.

Conclusion

We conclude that SA is well tolerated and has favorable outcomes in terms of cost-effectiveness, anesthesia time, surgical time, and hospital stay; therefore, SA should be considered for a greater number of lumbar spine surgeries, especially in low-middle income countries.

## Introduction

Lumbar spine surgeries are primarily performed under general anesthesia (GA). This is due to various reasons such as the surgeon’s preference, the anesthesiologist’s inclination, and the patient’s perception of the standard of care. Procedures that may be done in under two hours and do not have a significant risk of excessive blood loss may be performed under spinal anesthesia (SA). Appropriate patients for SA include those with low lumbar spine deformities (as surgical procedures for lower trunk require limited exposure and various studies have reported reduced complications), non-protuberant abdomens, intact cardiac status (as the prone position of surgery may compromise cardio-pulmonary systems) [1], and preference for daycare procedures. The appropriate anesthetic technique allows the rapid onset of anesthesia and easier reversal; spinal anesthesia does not require any external agent for reversal as it reverses spontaneously. Additional agents may reduce the time for reversal), maintenance of hemodynamic status, reduced stay in the recovery room, and reduced postoperative pain, nausea, and vomiting [2]. Tetzlaff et al. were among the first to report clinical experience with SA for elective spine surgery including perioperative complications, such as nausea and post-dural puncture headache (PDPH) [3]. These findings were repeated by Jellish et al. who further reported decreased intra-operative blood loss and time taken compared with patients undergoing similar procedures under GA [1]. Spinal anesthesia allows prolonged surgery time in a prone position without compromising the airway, avoids brachial plexus injury, avoids pressure necrosis of the face, reduces postoperative hospital stay, lowers the incidence of pulmonary complications [4] and perioperative cardiac ischemic events, and provides longer and adequate postoperative pain control [5].

SA is also more cost-effective than GA [6,7], which is a consideration, especially in countries like Pakistan where the annual gross domestic product (GDP) per capita in Pakistan was $1507.11 [8]. On the other hand, an average spine surgery costs $1000-$1,400; therefore, a cost-effective procedure is preferred, as most patients do not have health insurance.

As per the author’s knowledge, there is limited data from low-middle-income countries and South Asia on clinical experience with SA for lumbar spine surgeries. Munasinghe et al. reported the initial cases of two patients who underwent single-level, unilateral lumbar decompressions and discectomy under SA and discussed the importance of patient selection for SA in lumbar spine surgeries [9]. We present a case series of 45 patients who underwent lumbar spine surgeries under SA, and we correlate these parameters change with hospital stay and cost.

## Materials and methods

Ethical considerations

This study was approved by the Ethics Review Committee, Liaquat National Hospital and Medical College, Karachi, Pakistan. Informed consent was taken from the patients before the procedure. The data was anonymized so that patient identities remain confidential. The study is reported as per CARE guidelines.

Patients information

A total of 45 patients were included in our case series, among which 35.5% (n=16) were female and 64.5% (n=29) were male. No co-morbidities were present in 46.7% (n=21) patients, whereas 53.3% (n=24) had diabetes mellitus (n=9); hypertension (n=9); hypothyroidism (n=2); and ischemic heart disease (n=3). 46.7% (n=21) of patients were classified as American Society of Anesthesiologists (ASA) I 53.3% as ASA II, and there were no patients with severe systemic diseases. The mean BMI was 22.74±1.98 kg/m2. The most frequent presenting symptoms were backache (88.89%), pain in lower limbs (31.1% bilateral, 26.67% left, 28.89% right only), numbness in lower limbs (51.11% bilateral, 24.4% left, 24.45 right), and claudication (44.44%).

Diagnostic assessment

The visual analogue scale (VAS) was used to assess pain. 28.9% (n=13) patients had a VAS score of 9, 66.7% (n=30) had a score of 8, and 4.4% (n=2) had a score of 7 preoperatively.

The most common preoperative MRI findings included disc protrusion, narrowing of lateral recess, narrowing of neural foramina, compression of nerve roots, and decreased disc height. 51.1% (n=23) patients had MRI abnormalities in a single spinal level, 35.6% (n=16) in two levels, and 17.8% (n=8) in multiple spinal levels.

Surgical procedure

Following the diagnosis, all patients underwent conservative management of six weeks to two months which included analgesia with nonsteroidal anti-inflammatory drugs (NSAIDs), muscle relaxants, and/or gabapentin/pregabalin, along with adequate physiotherapy. Patients who failed to show a response to conservative management or had significant deficits affecting the quality of life were selected for surgical management. Patients were admitted on the day of the surgery following anesthesia assessment. Once the patients were shifted to the operating room, they were placed in the seated position and attached to ECG monitoring, noninvasive blood pressure, pulse oximetry, and a cutaneous temperature probe. SA was administered using lumbar puncture by a pencil-point needle, and 0.75% bupivacaine in an 8.5% dextrose solution. As bupivacaine is hyperbaric, it was administered one level above or below the deformity. No spinal canal stenosis was found in any patient and supplemental analgesia or sedation was not required. Motor block was assessed by asking the patient to raise his/her limb, whereas sensory block was assessed by asking for numbness in the limb. Once the block was effective, the patients were rolled into the supine position and allowed to rest until a stable spinal level was achieved. After an adequate block was documented, the patient was turned into the prone position on the operating table and placed in either the flexed or knee-chest position. Upper body and upper extremity positioning were modified to patient comfort, and the patients were placed on a pillow to allow easy breathing and to relieve anxiety about having their faces covered. Oxygen administration was provided by the nasal cannula and at a flow of 2 L/min. After the procedure, the propofol was not given, and the patients were rolled from the prone position onto the recovery bed and transferred to the PACU with O2 by nasal cannula. The time for spinal anesthesia was assessed from the beginning of the sensory block to the time the patients could move their limbs, and was compared with the time taken by GA. All the procedures were performed by the same surgical and anesthesia team.

The specific procedures are enlisted in Table 1. The levels of surgeries are enlisted in Table 2. Around 46.7% of patients (n=21) had a total spinal anesthesia time of 45 minutes, 48.9% (n=22) had a total spinal anesthesia time of 60 minutes, and 2 patients had 90 minutes. The total surgical time was 30 minutes in 46.7% of patients (n=21) patients, 48.9% (n=22) had a total surgical time of 45 minutes, and 2 patients had a surgical time of 60 and 80 minutes respectively.

**Table 1:** Surgical procedures

Surgical Procedure	Frequency (n=)	Percentage (%)
Interlaminar decompression	3	6.7
Laminectomy	42	93.3
Laminectomy + discectomy	18	40.0

**Table 2:** Levels of Surgeries

Single-Level Procedures	Level of Surgery	Frequency (n=)	Percentage (%)
	L3-L4	1	2.2
	L4-L5	11	24.4
	L5-S1	11	24.4
Two-level procedures	L3-L4, L4-L5	7	15.6
	L4-L5, L5-S1	9	20.0
More than two-level procedures	L3-L4, L4-L5, L5-S1	6	13.3

## Results

Outcomes

The total spinal anesthesia time was 45 minutes in 46.7% (n=21), 60 minutes in 48.9% (n=22), and 90 minutes in 4.4% (n=2). The total surgical time was 30 minutes in 46.7% (n=21), 45 minutes in 48.9% (n=22), 60 minutes in 2.2% (n=1), and 90 minutes in 2.2% (n=1). The total time of stay in the post-anesthesia care unit (PACU) was three hours in 46.7% (n=21), 48.9% (n=22) had a total time of stay of four hours, and only two stayed for five hours.

Common postoperative complaints included back pain in 46.7%; 17.8% (n=8) complained of both back pain and leg pain, 2.2% (n=1) complained of back pain and nausea, 11.1% (n=5) complained of leg pain, 2.2% (n=1) complained of nausea, and 20.0% (n=9) patients have no postoperative complaint.

The VAS scores were improved due to the surgical correction of the underlying pathology. Postoperatively, 46.7% (n=21) had a VAS score of 3, 46.7% (n=21) had a VAS score of 2, and 6.7% (n=3) had a score of 1; signifying improvement in a major symptom.

Day-care procedures were done in 71.1% (n=32). Around 22.2% of the patients (n=10) stayed in the hospital for one day, and 6.7% (n=3) stayed for more than one day. No complications occurred in 88.9% (n=40), whereas only 11.1% (n=5) complained of spinal headaches or PDPH which was reported by the patients in their first follow-up. The patients were given instructions about potential complications such as PDPH along with their management.

The total hospital cost was $360 for 48.9% (n=22); $450 for 33.3% (n=15); and $540 for 17.8% (n=8) patients. The cost was lower than that of surgeries done under GA which cost $900-$1125.

## Discussion

We report the initial case series of SA for lumbar spine surgeries from a single institution in Pakistan. Our experience indicates that SA is safe, takes lesser time, and has minimal to no complications in lumbar spine surgeries.

Pierce et al. conducted a retrospective analysis comparing SA to GA for laminectomy and/or diskectomy spinal surgery and reported lower operative time, blood loss, total anesthesia time, the total time for surgery, and duration of hospital stay, however, a longer stay in the PACU was reported [10]. Attari et al. conducted a randomized controlled trial comparing SA to GA and along with the previously reported findings, intraoperative maximum blood pressure and heart rate changes were also reported to be lower in the SA group. Additionally, surgeon satisfaction was greater and postoperative mean VAS was significantly lower in the SA Group [11]. Perez-Roman et al. conducted a meta-analysis of 3709 patients undergoing SA for lumbar discectomies and laminectomies. Also, they concluded that patients who underwent SA had decreased total anesthesia time, operative time, and post-operative complications [12]. Meng et al. replicated these findings [13].

Munasinghe et al. reported cases of two unilateral L4-5 decompression and discectomy with satisfactory surgical outcomes. They discussed a lower rate of postoperative complications and the importance of selecting appropriate cases. Multi-level canal stenosis or complicated cases require bilateral dissections which prolonged operative time thus single or two-level decompression should undergo SA. Other unsuitable candidates include obese patients, the elderly, multiple comorbidities, and children who might not cooperate with prolonged prone positioning [9]. SA however, has proven to be effective and safe for geriatric populations, including those with degenerative conditions [14], and even for patients older than 84 years and for surgeries lasting up to three-and-half hours [15]. Breton et al. compared GA and SA in complex procedures such as complex instrumented fusion and reported similar results to the reported literature [16]. SA is also safe in high-risk patients as demonstrated by Patil et al. in a retrospective study [17].

Lee et al. listed and compared the advantages of both regional and general anesthesia and recommended that regional anesthesia should be the standard of practice in lumbar spine surgeries [18]. West et al. demonstrated that SA can be safely combined with a microscope- and loupe-assisted approaches to lumbar surgeries, and SA can be adopted as a standard without any significant learning curve [19].

Our study had a limited population as this study was based on a single center. The surgeon satisfaction and patient satisfaction were not quantitatively assessed. The postoperative follow-up was limited as the patients had to travel long distances for it. We recommend conducting lumbar spine surgeries under SA and further studies in SA in the developing world.

## Conclusions

Even though lumbar spine surgeries under GA are the norm, this case series provides the feasibility of SA for lumbar spine surgeries. SA is well tolerated and has favorable outcomes in terms of anesthesia time, surgical time, and hospital stay. Our study replicates the findings of the previous literature comparing GA and SA.
